# Supplementary material for: Catalyst-Free Cardanol-Based Epoxy Vitrimers for Self-Healing, Shape Memory, and Recyclable Materials
Source: Polymers (Basel). 2024 Jan 23;16(3):307. doi: 10.3390/polym16030307 (PMC10857227; doi:10.3390/polym16030307)
Supplement: Supplementary file 1 [file polymers-16-00307-s001.zip › polymers-2746024-supplementary.pdf]

Supporting Information

Catalyst-Free Cardanol-Based Epoxy Vitrimers for Self-Healing, Shape Memory, and Recyclable Materials

Yu Zhu [1, 2], Wenbin Li [1], Zhouyu He [2], Kun Zhang [1], Xiaoan Nie [1], Renli Fu [2, *] and Jie Chen [1, *]

[1] Key Laboratory of Biomass Energy and Material, Jiangsu Province, Institute of Chemical Industry of Forest Products, Chinese Academy of Forestry, Nanjing 210042, China

[2] College of Materials Science and Technology, Nanjing University of Aeronautics and Astronautics, Nanjing 210016, China

* Correspondence: renlifu@nuaa.edu.cn (R.F.); jiechen@icifp.cn (J.C.)

Summary of Content:

Number of pages: 13

Number of figures: 12

Number of schemes: 1

Figure S1 ^{1}H NMR spectrum of CD……………………………………………………..S1

Figure S2 ^{1}H NMR spectrum of CGE……………...………………………………S2

Figure S3 ^{1}H NMR spectrum of CAP……………...………………………………S3

Figure S4 ^{1}H NMR spectrum of CAPA. ……………...……………………………S4

Scheme S1 Synthesis of cardanol polyacid…...………………………..…………S5

Figure S5 FTIR spectrum of CDPA……………...……….……………...………S6

Figure S6 ^{1}H NMR spectrum of CDPA..……………...……….……….....………S7

Figure S7 DSC thermograms of DGEBA-CAPA systems...……....……….....………S8

Figure S8 DSC thermograms of DGEBA-CDPA systems. ……………...………….. S9

Table S1 Specific ratio of epoxy monomer and curing agent…...………...…………S10

Figure S9 FTIR spectra of A (DGEBA-1CAPA), B (DGEBA-0.75CAPA), C (DGEBA-0.5CAPA) and D (DGEBA-0.25CAPA)…...…………………………..……....…S11

Figure S10 Self-healing of DGEBA-1CDPA with different time at 180 °C....…….. S12

Figure S11 Comparison of FT-IR spectra of DGEBA-1CAPA initially and after self-healing……………...……...…...………..…………………….……………...S13

Figure S12 Reprocessed DGEBA-CDPA. ……………...…………………..…....…S14

Figure S13 Mechanical properties of reprocessed DGEBA-CAPA. …………...……S15

Cardanol(CD)

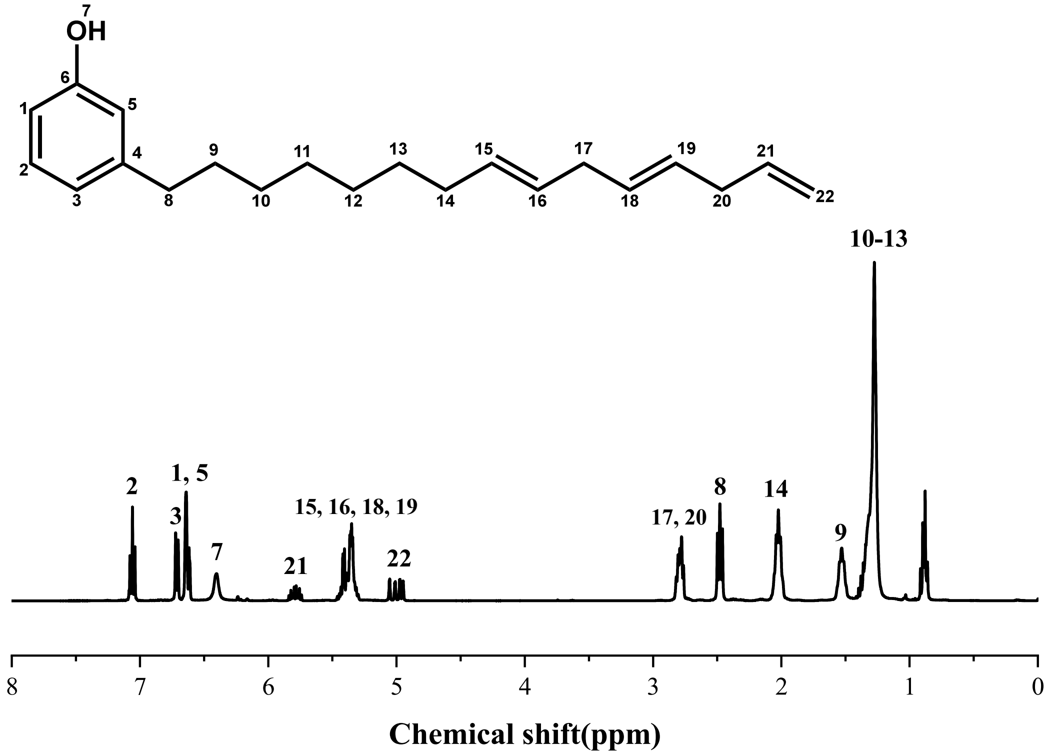

Figure S1. ^{1}H NMR spectrum of CD.

Cardanol Glycidyl Ether (CGE)

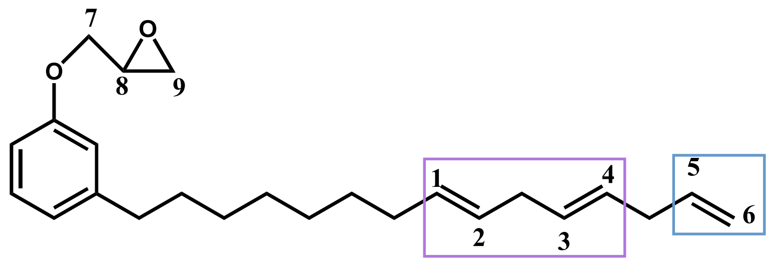

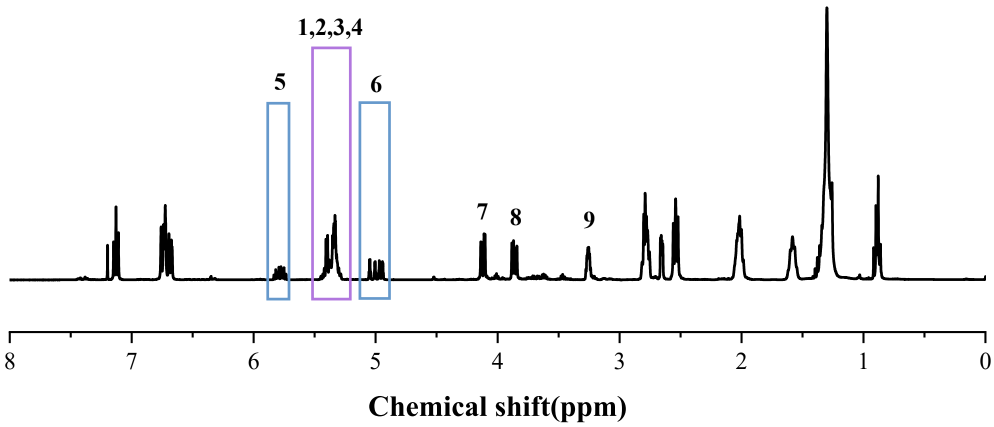

Figure S2. ^{1}H NMR spectrum of CGE.

Cardanol Amine Polyol (CAP)

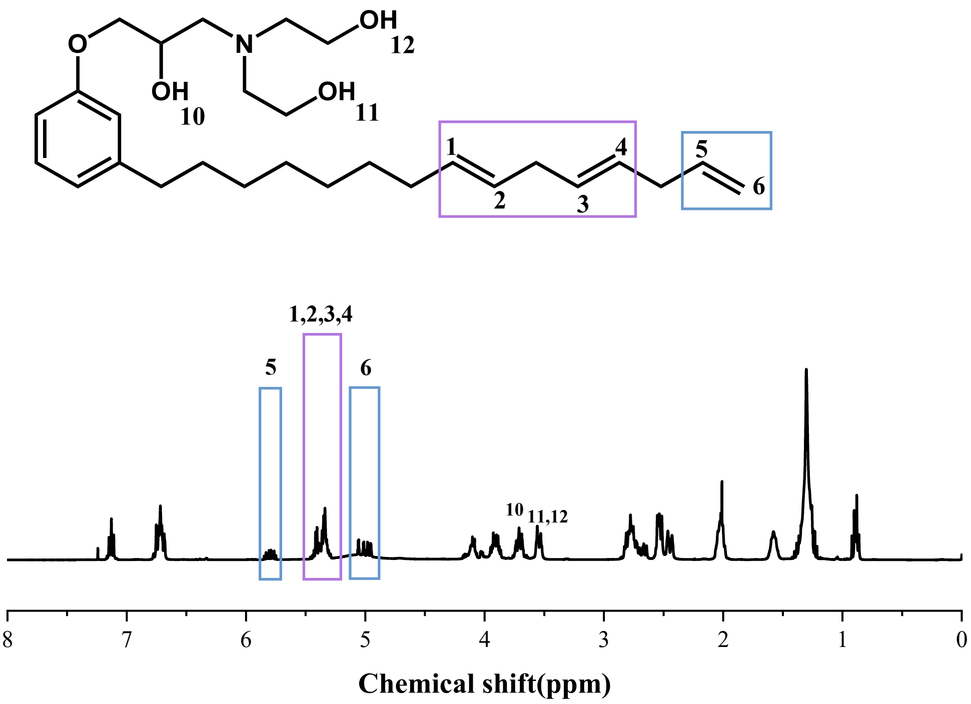

Figure S3. ^{1}H NMR spectrum of CAP.

Cardanol amine polyacid (CAPA)

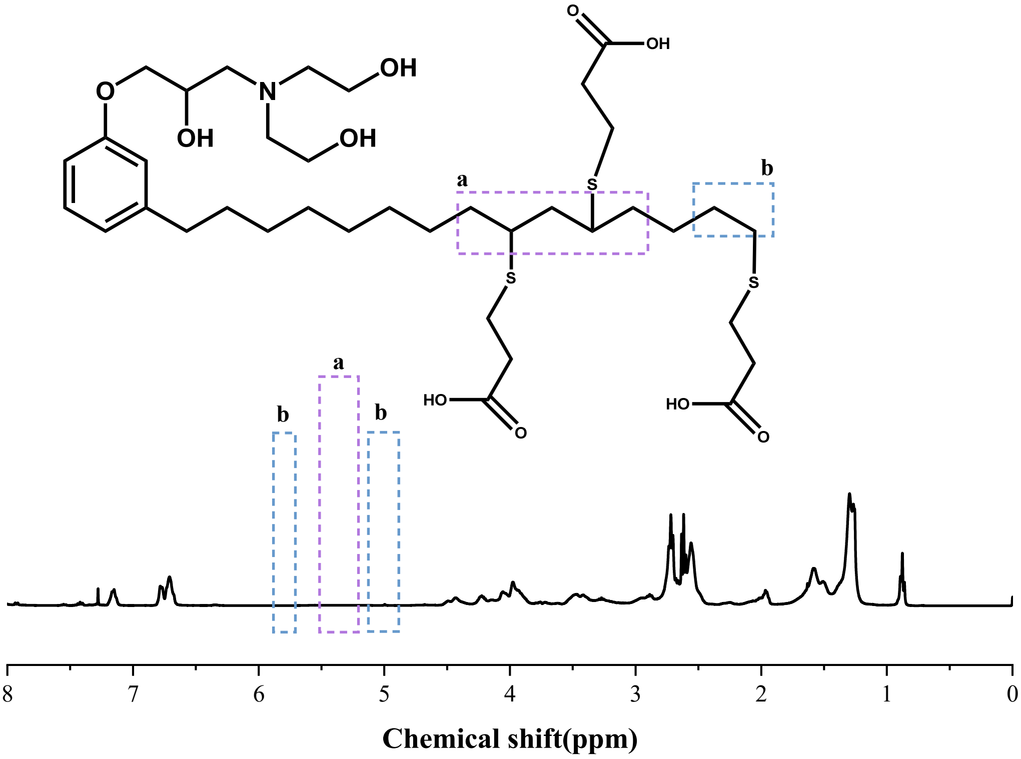

Figure S4. ^{1}H NMR spectrum of CAPA.

1.1. Synthesis of Cardanol polyacid.

CD (0.08 mol, 24.16 g), MPA (0.24 mol, 25.44 g) and photo initiator 1173 (2 wt%, 0.99 g) were added into quartz glass beaker. The mixture was stirred well and reacted for 12 h in the case of ultraviolet (UV)-light. The wavelength range of ultraviolet light is 300-350 nm. The goal products were obtained after removing excess MPA by rotary evaporation at 80 °C, named as CAPA (acid value: 275 mg KOH g^{-1}).

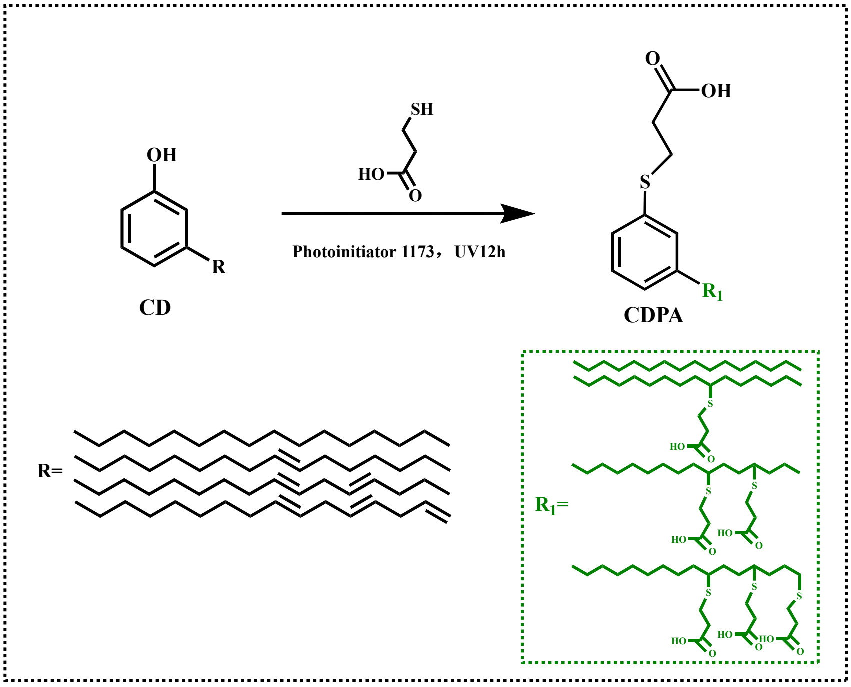

Scheme S1. Synthesis of cardanol polyacid.

1.2. Characterizations of cardanol polyacid.

The ^1H nuclear magnetic resonance (^1H NMR) and fourier transform infrared spectrometry (FTIR) spectra were used to determine the molecular structures of CDPA. As shown in Figure S5, the peaks of C=C at 3008 and 878 cm^{-1} disappeared and a broader peak of carboxyl group peak appeared in the spectrum of CDPA, which indicates the successful preparation of CDPA. Due to the high activity of the phenolic hydroxyl group, it can undergo a dehydration reaction with mercaptopropionic acid, so

the peak of the phenolic hydroxyl group at 3337 cm^{-1} disappeared. Figure S6 represents the ^{1}H NMR spectra of CDPA, the double bond signals at 5.2-5.4, 5.7-5.8, and 4.9-5.0 ppm almost completely disappeared. In addition, the disappearance of the phenolic hydroxyl signal at 6.3-6.4 ppm also proved that the phenolic hydroxyl was reacted.

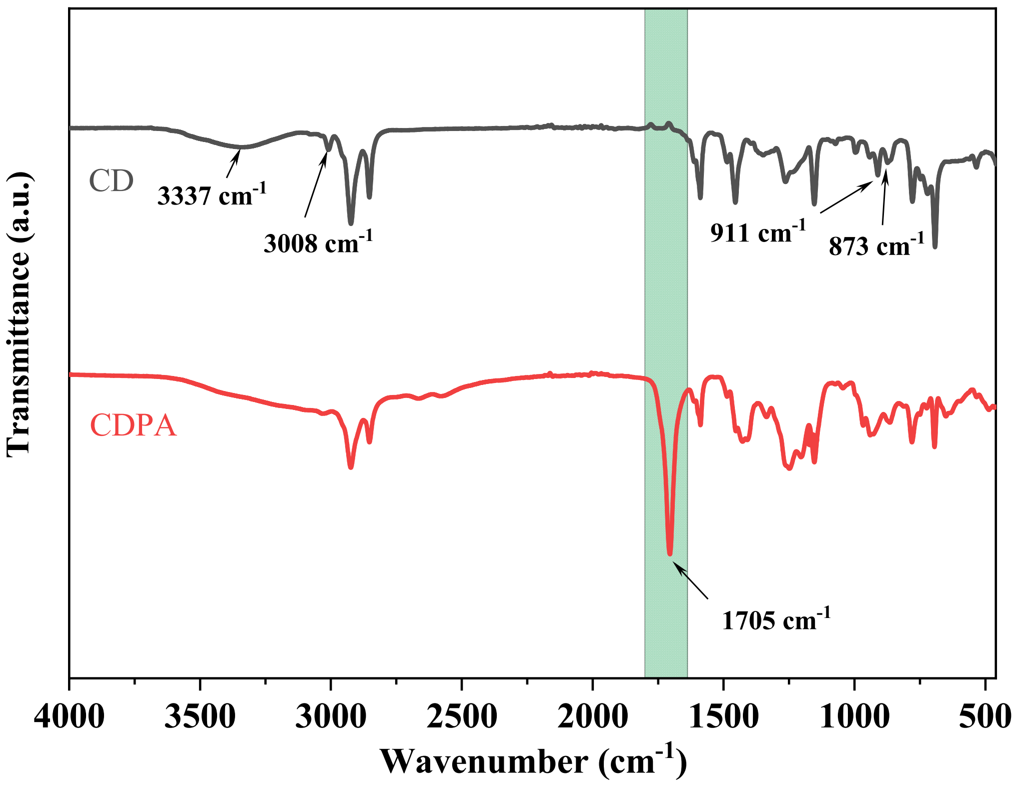

Figure S5. FTIR spectrum of CDPA.

Cardanol polyacid (CDPA)

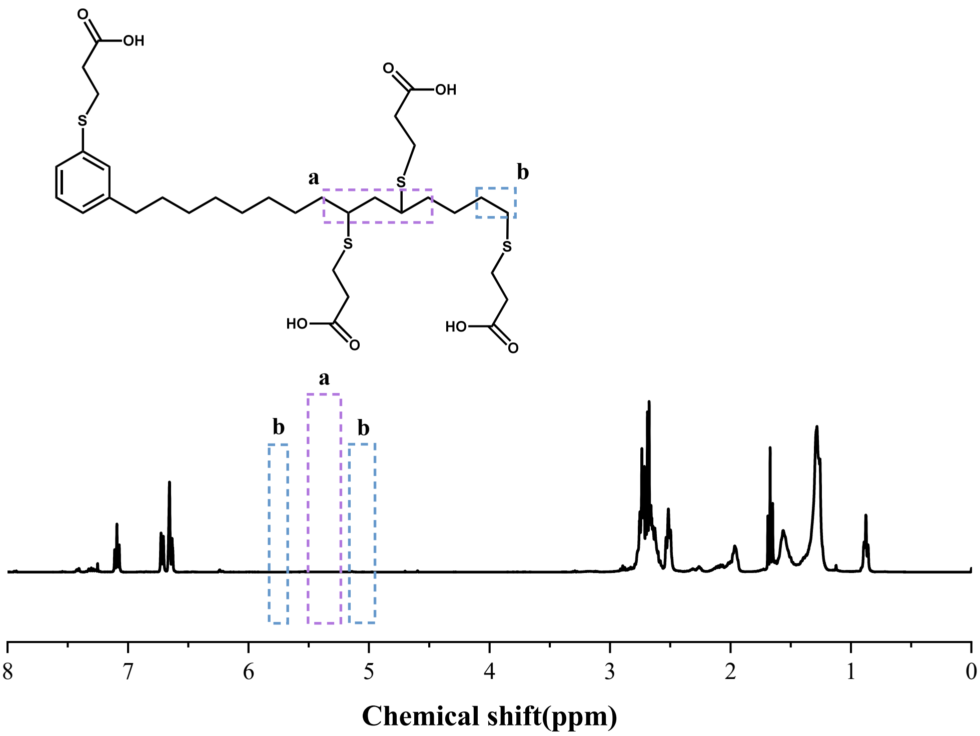

Figure S6. ^{1}H NMR spectrum of CDPA.

2.1 Curing behavior of DGEBA-CAPA vitrimers

In order to study the curing behavior of DGEBA-CAPA, the exothermic peak of the curing process was measured by DSC. As shown in Figure S7, the DGEBA-CAPA system with carboxyl-epoxy ratios of 0.75, and 1.0 have an exothermic peak around 145 °C in the absence of catalyst, indicating the occurrence of a curing reaction. DGEBA-0.5CAPA and DGEBA-0.25CAPA have two exothermic peaks, representing the curing process of epoxy group and carboxyl group, epoxy group and hydroxyl group, respectively. Therefore, determining the terminal curing behavior of the DGEBA-CAPA system at 160 °C for 4 h and 180 °C for 1 h can ensure its complete curing. In addition, the curing process of the DGEBA-CDPA system is similar to it, so a single sample is selected for description (Figure S8).

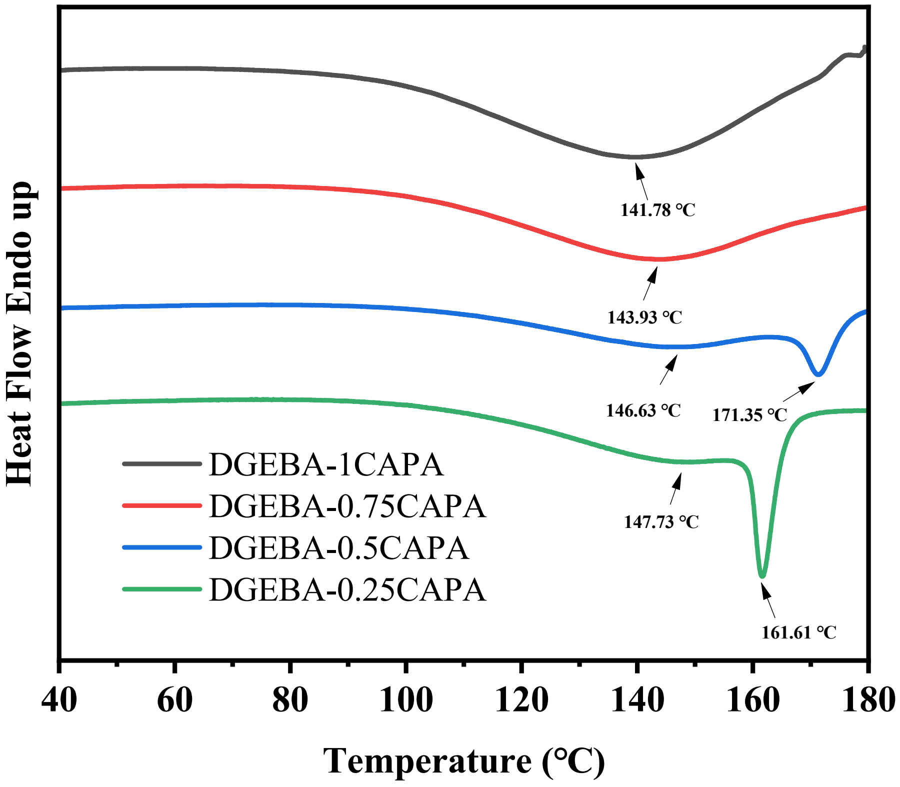

Figure S7. DSC thermograms of DGEBA-CAPA systems.

2.2 Curing behavior of DGEBA-CDPA vitrimers.

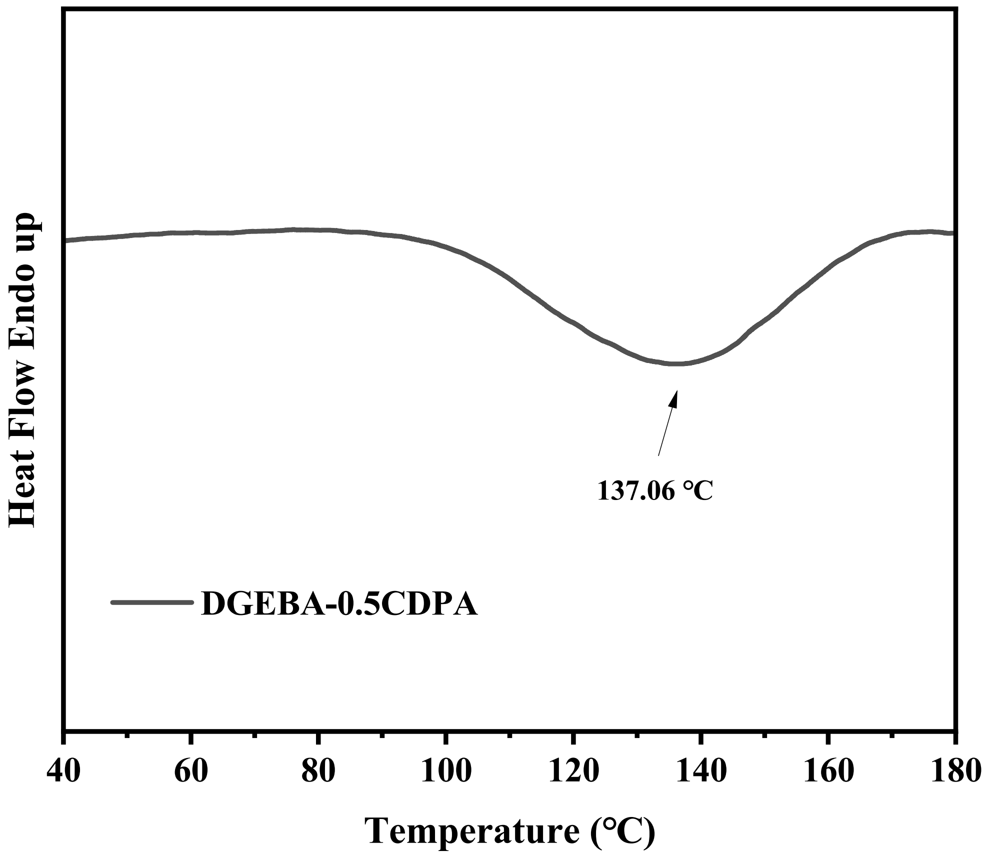

Figure S8. DSC thermograms of DGEBA-0.5CDPA systems.

Table S1 Specific ratio of epoxy monomer and curing agent.

Samples	epoxy monomer (g)	curing agent (g)	DMP-30 (g)
DGEBA-1CAPA	20.0	33.6	-
DGEBA-0.75CAPA	20.0	25.2	-
DGEBA-0.5CAPA	20.0	16.8	-
DGEBA-0.25CAPA	20.0	8.4	-
DGEBA-0.5CDPA	20.0	10.4	0.03

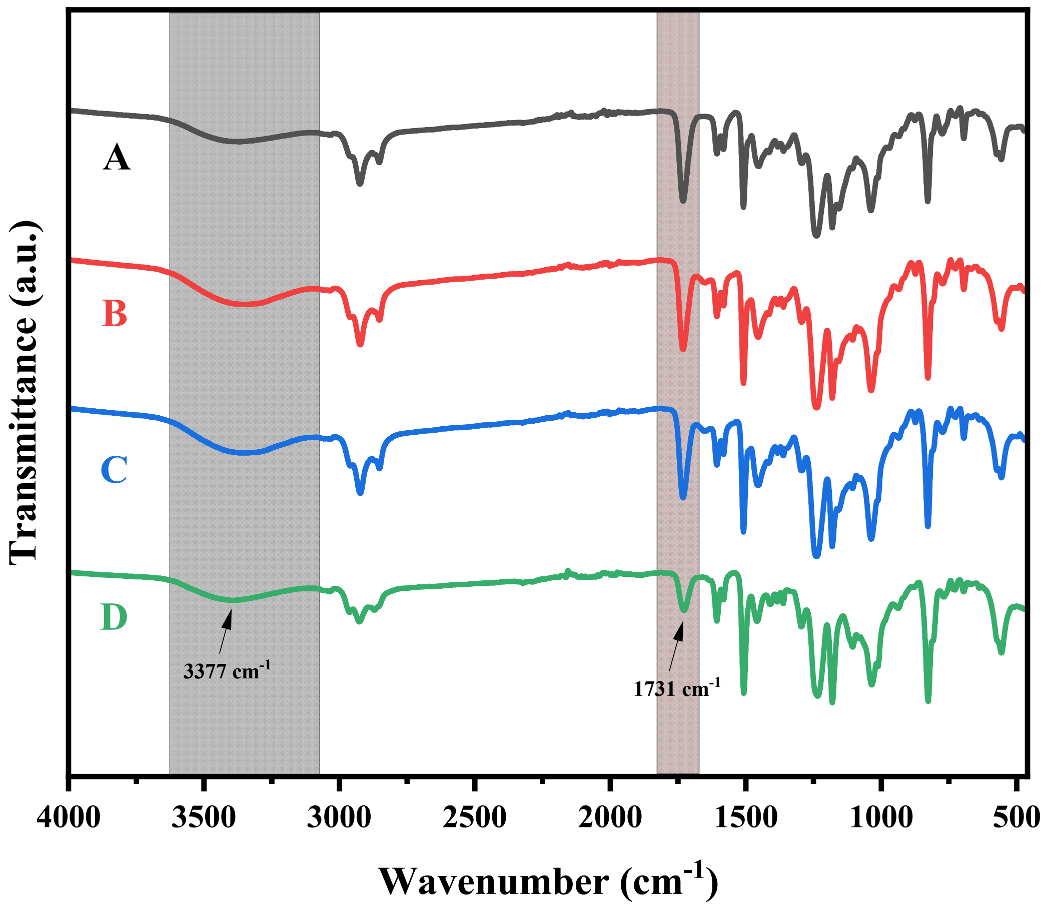

Figure S9. FTIR spectra of A (DGEBA-1CAPA), B (DGEBA-0.75CAPA), C (DGEBA-0.5CAPA) and D (DGEBA-0.25CAPA).

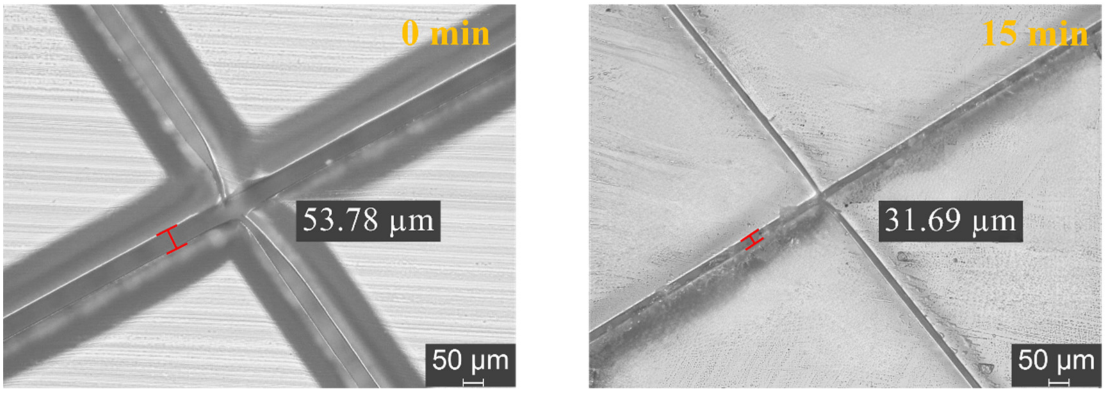

Figure S10. Self-healing of DGEBA-1CDPA with different time at 180 °C.

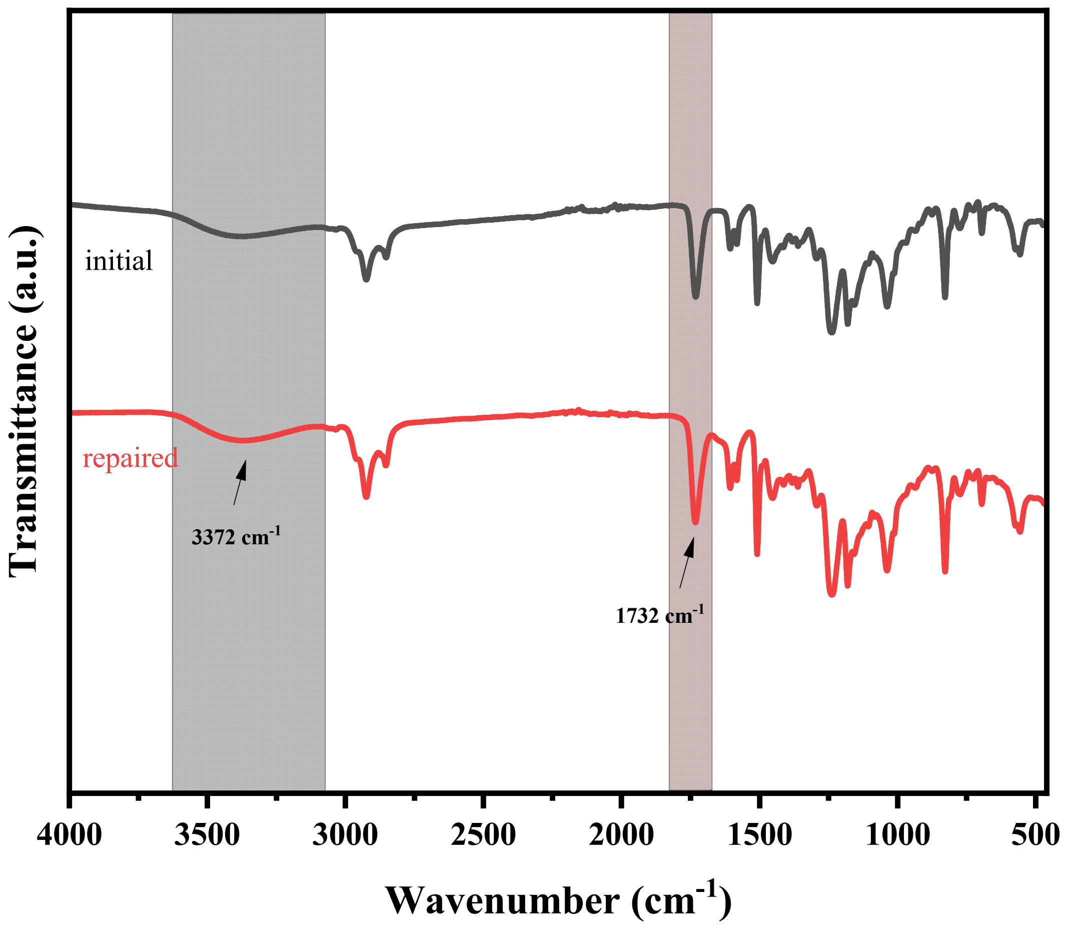

Figure S11. Comparison of FT-IR spectra of DGEBA-1CAPA initially and after self-healing.

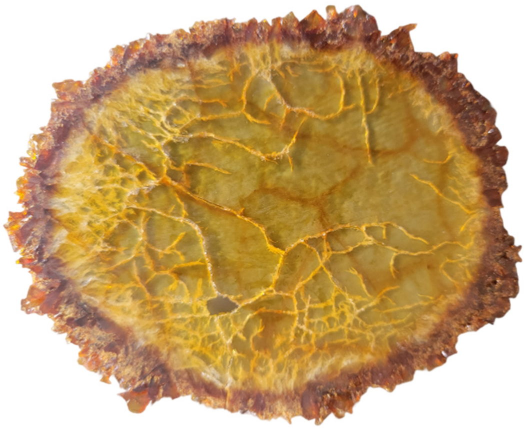

Figure S12. Reprocessed DGEBA-1CDPA.

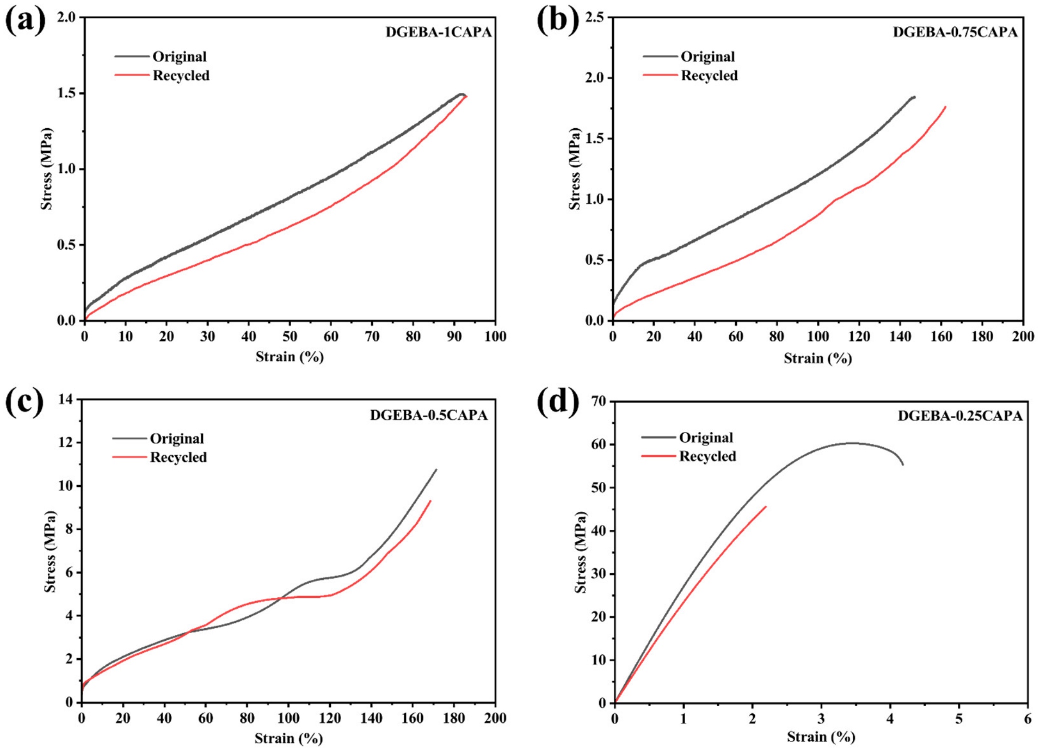

Figure S13. Mechanical properties of reprocessed DGEBA-CAPA.
